# Dendritic cells in multiple myeloma: from immune evasion to therapeutic potential

**DOI:** 10.3389/fimmu.2025.1575509

**Published:** 2025-04-17

**Authors:** Melanie Andrea Jordan, Johannes Morschl, Stella E. Autenrieth

**Affiliations:** Dendritic Cells in Infection and Cancer, German Cancer Research Center, Heidelberg, Germany

**Keywords:** dendritic cells, multiple myeloma, immune evasion, DC vaccine therapy, tumor microenvironment

## Abstract

Multiple myeloma (MM) is a type of hematologic cancer characterized by the uncontrolled clonal expansion of plasma cells in the bone marrow (BM). This leads to significant dysfunction and suppression of the immune system in affected patients. Myeloma cells employ sophisticated strategies to manipulate immune and non-immune cells, evading immune surveillance and enhancing their survival. One key factor in this evasion is the disruption of dendritic cell (DC)-mediated immune mechanisms. Extensive evidence indicates that in the presence of myeloma cells, DC numbers are notably reduced, and their phenotype and function are altered, impairing their ability to present antigens and activate robust T-cell responses effectively. Despite rapid advances in MM treatment, with promising strategies such as DC-based vaccines being already achieved, DC dysfunction remains a substantial hurdle, associated with or contributing to poor therapeutic outcomes, disease relapse, and MM’s persistence as an incurable disease. To address these challenges, it is essential to understand the intricate mechanisms through which myeloma cells transform DCs into their “accomplices,” undermining immune responses. This review comprehensively summarizes the current understanding of the role of DCs in MM. Additionally, it evaluates the potential of DCs in anti-MM immunotherapy, discussing persistent challenges and highlighting emerging perspectives that may lead to promising breakthroughs for improved patient outcomes.

## 1 Introduction

MM is a B-cell malignancy characterized by the uncontrolled proliferation of plasma cells, primarily within the BM. It is the second most common hematologic cancer, accounting for 10–17% of cases, and predominantly affects individuals over 40, with a median diagnosis age of 65. Men are at a higher risk than women (1).

MM often progresses from two asymptomatic precursor stages: monoclonal gammopathy of undetermined significance (MGUS) and smoldering MM (SMM). MGUS has <10% clonal plasma cells in the BM with a 1% annual progression risk (2), while SMM has >10% clonal plasma cells and a 10% annual progression risk (3). Clinical symptoms are summarized by the CRAB criteria: hypercalcemia, renal dysfunction, anemia, and bone lesions (4).

A hallmark of MM is severe immunodeficiency. Myeloma cells evade immune responses by impairing DCs, critical for activating T cells. This immune suppression facilitates disease progression and limits the efficacy of therapies, including DC-based vaccines. Despite advancements in treatment, MM remains incurable, with a median survival of 5–7 years (5). Understanding DC interactions within the tumor microenvironment is essential for developing effective immune-based therapies.

## 2 DC in health & disease: a brief overview

DCs are pivotal antigen-presenting cells that link innate and adaptive immunity by activating T-cell responses. They process and present antigens via MHC molecules, enabling T-cell priming and differentiation. Human DCs are classified into three main subsets: conventional DCs (cDCs), plasmacytoid DCs (pDCs), and monocyte-derived DCs (moDCs) (6).

cDCs are divided into cDC1s, cDC2s, and DC3s according to transcriptional and functional properties. cDC1s express markers such as XCR1, DNGR-1 (CLEC9A), CD141 (BDCA3), and CADM1 and are specialized in cross-presenting antigens to CD8^+^ T cells, promoting cytotoxic responses (6). Ex vivo studies demonstrated their ability to stimulate naïve CD4 T cells into Th1 and Th2 cells (7–9).

cDC2s are more heterogeneous and express CD11b, CD1c (BDCA1), and SIRPα (CD172a). cDC2s promote CD4^+^ T cell activation and differentiation into Th1, Th2, Th17 and T follicular helper cells (10).

Subsets of cDC2s include DC2s and DC3s, the latter sharing features with monocytes but developing differently (11, 12).

pDCs play a critical role in antiviral immunity by producing type I interferons and expressing markers such as CD123, BDCA2 (CD303), and BDCA4 (CD304) (13). MoDCs arise during inflammation and share phenotypic similarities with cDCs, but they can be distinguished by CCR2 and Fcγ receptors.

All DC subsets originate from hematopoietic stem cells in the BM. They develop through granulocyte-monocyte DC precursors (GMDPs) into common DC progenitors (CDPs) before differentiating into pre-cDCs or pre-pDCs. While pre-cDCs migrate to peripheral tissues to mature into cDCs, pDCs complete their differentiation in the BM (14).

Upon activation by inflammatory signals or pathogens, immature DCs mature, upregulating MHC and co-stimulatory molecules like CD80, CD86, and CD40. Mature DCs migrate to lymphoid organs to initiate immune responses (15). However, under oncogenic or septic conditions, DC functions can be impaired, promoting disease progression. Understanding these subsets is crucial for advancing immunotherapy and cancer treatment (16, 17).

## 3 Myeloma immune escape: role of DCs

Immune surveillance is crucial for the immune system to recognize and eliminate cancer cells before tumor formation. This concept ties into immunoediting, which outlines the dynamic between the immune system and tumors. Cancer immunoediting includes three phases: elimination, equilibrium, and escape, each marking a specific interaction stage (reviewed in detail in (18–20)).

In the premalignant stages of MM, particularly during MGUS and SMM, the immune system initially targets emerging malignant clones. However, as MM progresses, specific genetic alterations may allow certain tumor subclones to persist. This persistence creates a fragile balance where some myeloma cells are eradicated while more resilient cells remain in a dormant state maintained by the immune system. It is proposed that when the immune system can no longer sustain this delicate equilibrium, it leads to the uncontrolled proliferation of malignant cells (21), marking the transition from MGUS and SMM to active MM (reviewed in detail in (5)). MM genetic events include chromosomal abnormalities like trisomies, IgH translocations, somatic mutations, and secondary cytogenetic abnormalities like Del(17p) (22).

### 3.1 The tumor microenvironment

The tumor microenvironment (TME) is a complex ecosystem around a tumor, comprising cellular elements like cancer, stromal, and immune cells and non-cellular components like blood vessels, the extracellular matrix (ECM), and secreted factors. It is dynamic and crucial for tumor growth, progression, metastasis, and treatment response (23). Evidence shows that myeloma cells employ various mechanisms to suppress antitumor immunity while aiding their survival (24). These mechanisms primarily involve interactions with immune and non-immune cells in the BM microenvironment (BMME), which are related to MM risk and severity (25–29). While the overall composition influences MM progression, the specific roles of individual immune cells need further exploration, though DCs are recognized as key players in MM’s progression (30).

### 3.2 DCs in the MM-microenvironment

DCs activate T cells and infiltrate tumors, making them vital for immune responses in various cancers, including MM. However, the TME often impedes their functions, weakening anti-tumor responses. In the TME, DCs become dysfunctional, compromising immune responses due to immunosuppressive TME traits that affect maturation, migration, and effector functions (31, 32). This dysfunction impairs antigen capture and downregulates costimulatory molecule expression. Conventional type 1 DCs (cDC1s) are essential for generating anti-tumor responses by cross-presenting tumor antigens from necrotic cells (33). Also, stimulatory DC infiltration correlates with better prognosis and immunotherapy response (34). Recent studies found a universal mature DC population within the TME, but their development and functions are unclear. This group, called “mregDCs” or “LAMP3^+^ DCs,” shows similarities and heterogeneity due to environmental factors (35).

Extensive evidence emphasizes DCs’ central role in MM pathophysiology. Instead of fostering effective immune responses, DCs often become impaired (36–38), promoting MM cell survival, tumor growth, and immune evasion (24, 30). DC dysfunction stems from various molecular mechanisms involving direct interactions between myeloma cells and DCs or high levels of cytokines, especially IL-6, in the TME (37). This results in the aberrant activation of key signaling pathways and advances disease progression.

### 3.3 Myeloma-DC cellular interaction

Previous studies show that DCs enhance myeloma cell survival in the BMME through direct interactions that activate APRIL and BAFF signaling pathways, essential for plasma cell growth. APRIL and BAFF, produced by myeloid cells like DCs and monocytes, regulate normal B lymphocyte and plasma cell survival through BCMA or TACI receptors. APRIL and BAFF levels rise significantly in MM, with their receptors overexpressed on myeloma cells (39, 40). While BCMA is typically expressed more than TACI on these cells (41), Kukreja et al. and Chauhan et al. found that moDC/pDC-myeloma interactions mainly promote BAFF/APRIL signaling via TACI, aiding malignant plasma cell growth, respectively (24, 30). Although mechanisms are not fully understood, studies confirm blocking the BAFF/APRIL–TACI interaction inhibits DC-mediated tumor clonogenicity in myeloma cells (24, 30). However, newer studies show that pDCs from MM patients show higher BCMA levels than myeloma cells, suggesting BCMA on pDCs may be vital for supporting myeloma cell survival (42).

### 3.4 Cytokine-mediated effects on DCs

In addition to directly interacting with MM cells, DCs facilitate immune evasion in MM, stimulated through immunosuppressive cytokines, including transforming growth factor-β1 (TGF-β1), vascular endothelial growth factor (VEGF), IL-6, and IL-10. These are primarily secreted by myeloma and BM stromal cells during their interactions (43). The cytokine signaling activates key cell growth and survival pathways, impairing DCs (44). For instance, IL-6 binding to its receptors activates Janus kinases (JAKs) and STAT proteins, particularly STAT3, which diminishes DC function (45–48).

## 4 DC impairments in MM

**Figure 1:**
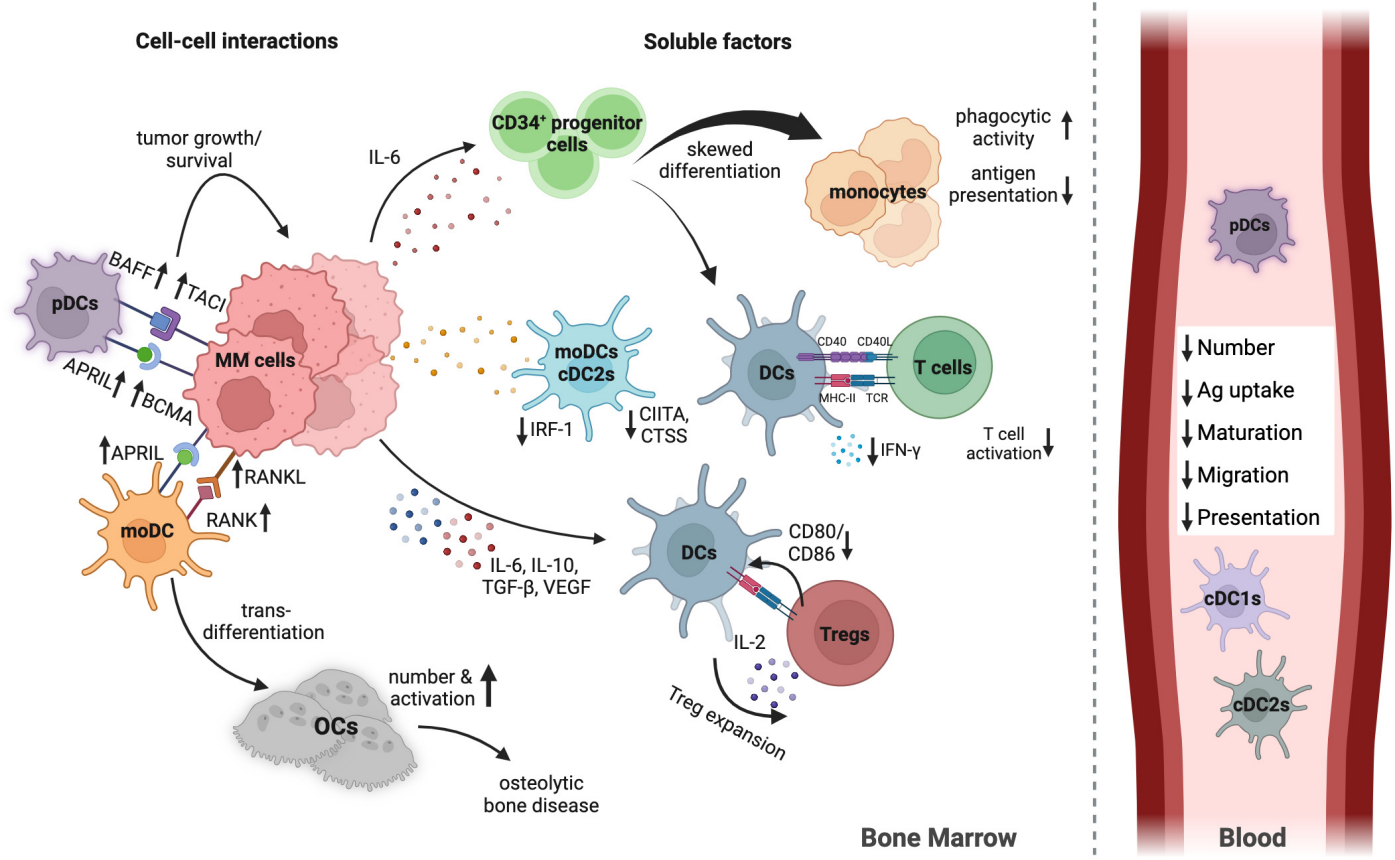
Human DC in MM BMME. This overview outlines the main mechanisms of DC impairment in the MM TME. Not all mechanisms are detailed for simplicity. MM cells limit the number, differentiation, maturation, and function of various DC subsets by secreting inhibiting cytokines and engaging directly with DCs. Elevated IL-6 shifts differentiation from CD34^+^ progenitor cells to monocytes rather than DCs. IL-10, VEGF, and TGF-β impair DC migration and maturation. DCs and MM cells communicate via TACI and APRIL, promoting tumor growth. The interaction between them increases RANKL expression on MM cells, while DCs express RANK, triggering DC transdifferentiation into osteoclasts, contributing to MM-related bone disease. mRNA expression of IRF-1, CIITA, and CTSS in moDCs and cDC2s decreases in MM, impairing maturation and antigen presentation pathways. pDCs are less effective at activating T cells and stimulating IFN-γ due to immunosuppressive factors in the TME. Additionally, DCs facilitate Treg expansion through direct contact with T cells and low CD80/CD86 co-stimulatory molecule expression. In the peripheral blood DC subsets are reduced in numbers, combined with less expression of migration and maturation markers, and reduced antigen uptake and presentation capacity. Created with Biorender.com.

Strong evidence shows DCs are impaired in MM, prompting numerous studies on their numbers, phenotype, and function throughout progression (36). Despite some contradictions in findings, consistent observations include a significant decrease in DCs and cytokine-mediated changes impacting DC differentiation, maturation, and function (**Figure 1**).

### 4.1 Reduced DC numbers in the periphery of MM patients

DCs comprise 0.1-2.0% of peripheral blood mononuclear cells. Still, studies show a significant decrease in both cDCs and pDCs in MM patients compared to healthy individuals (49, 50). For instance, Do et al. noted a 50% reduction in blood DCs among MM patients (51). Similar DC depletion has been observed in other cancers and infections (52–57). Ratta et al. found that peripheral blood DCs are stable regardless of disease stage, with a trend for lower PBDCs in patients with more significant tumor burdens (37). Studies show fewer DCs in advanced disease stages in MM patients. While the reasons for these discrepancies are unclear, factors like sample collection variability and chemotherapy could affect DC counts. Nevertheless, there is a clear trend of declining DC numbers as MM progresses, yet studies on specific DC subsets in untreated MM patients are lacking.

During DC development, they differentiate from CD34^+^ hematopoietic stem cells through several stages. High IL-6 levels in the TME may skew differentiation towards monocytes instead of DCs, at least in GM-CSF cultures, potentially explaining reduced blood DC numbers in MM patients (37).

Research indicates increased pDC abundance in the BMME (24) but diminished in the PB (36). Moreover, MM progression from MGUS correlates with increased cDCs and pDCs in the BM, which can contribute to immunosuppression and tumor growth. However, they did not distinguish cDC1s from cDC2s, focusing only on CD141^+^ cDC1s, which have distinct roles and are fewer than cDC2s. Consequently, their response in MM may vary significantly (50).

A recent analysis of DC subsets post-BCMA CAR-T therapy revealed increased proportions of all cDC types except pDCs in the BMME (58). However, the variability in the patient cohort limits the findings. Overall, studies confirm significant reductions in conventional and pDCs in MM patients. These DCs impact T-cell responses and foster immune tolerance through immature DC interactions with myeloma cells.

### 4.2 Impaired DC activation and maturation in MM

DC number alterations due to immunosuppressive cytokines IL-6, IL-10, VEGF, and TGF-β1 in the TME of MM link to significant phenotypic changes in these cells. These changes indicate functional deficiencies affecting DC maturation, antigen presentation, and T-cell priming. Key markers like HLA-DR, CD40, CD80, and CD86 show lower expression on PB myeloid and pDCs from MM patients than healthy donors (36–38). Despite high phagocytic activity, antigen presentation capacity is low (37). Blocking IL-6 with anti-IL-6 antibodies does not fully restore this function, suggesting additional factors impair DC functions in MM (37). Brown et al. showed that IL-10 and TGF-β from myeloma cells hinder DC maturation by affecting CD80 and CD86 expression, which can be reverted using either IL-12 or IFN-γ (59, 60). DC maturation involves complex processes for efficient tumor antigen handling, regulated by interferon regulatory factor 1 (IRF-1). IRF-1 controls MHC class II transactivator (CIITA) for HLA class II expression and cathepsin S (CTSS) activity that facilitates antigen peptide binding (61). Recent findings by Jiang et al. indicate IRF-1 expression is low in cDC2s and mo-DCs of MM patients, leading to decreased CIITA and CTSS levels, harming maturation and antigen presentation capacity (62).

Additionally, Brimnes et al. reported reduced CCR5 and CCR7 expression in cDCs and pDCs during MM (36). CCR5 mediates immature DC migration to inflammation sites, while CCR7 indicates DC maturation, guiding them to lymph nodes for T cell priming (63). Impaired CCR5 and CCR7 expression further highlights defective DC maturation in MM. Shinde et al. linked impaired CCR7 migration to increased IL-6 levels and p38 activation in the MM TME. These observations reveal dysregulation in DC maturation in MM, suggesting complex mechanisms behind the immature DC phenotype warranting further exploration (64).

### 4.3 Impaired T cell priming by pDCs and moDCs in MM

DCs are essential for the immune system to effectively target and eliminate myeloma cells. They must maintain a balance of hematopoiesis, migration, maturation, and antigen processing to prime T-cell responses and expand T-cell clones that recognize and destroy malignant cells. However, studies show deficiencies in MM’s DC differentiation, maturation, and migration. This leads to a loss of tumor antigenicity and reduced T-cell priming, allowing MM to evade the immune response (24, 36, 37, 51, 60). In human MM models, pDCs and moDCs play critical roles, while cDCs remain underexplored. In mixed lymphocyte reactions, PB myeloid and pDCs from MM patients show reduced T cell proliferation. Brimnes et al. first note that they stimulate IFN-γ production by T cells much less than DCs from healthy individuals (36).

Similarly, moDCs from MM patients exhibit reduced co-stimulatory marker expression and impaired cytokine expression, like less IL-12p70 and increased IL-10, leading to reduced T cell activation and proliferation (64), indicating a tolerogenic DC phenotype.

Studies also highlight the DC-T cell interaction fostering Treg differentiation in the MM TME (65). In inflammatory conditions, DCs co-cultured with T cells expand functional Tregs, dampening T-cell responses (66, 67). Tregs may inhibit co-stimulatory molecule expression on DCs and reduce their pro-inflammatory cytokine release (68). These interactions suggest that expanding Tregs might exacerbate DC dysfunction in MM, as also shown in a mouse model of MM (69), although the precise mechanisms require further investigation.

Taken together, these studies indicate a tolerogenic DC phenotype in MM. MM-DCs exhibit functional traits similar to mregDCs (e.g., immunosuppressive cytokine profiles, impaired T cell priming), suggesting possible parallels. However, no direct evidence exists regarding the involvement of mregDCs or Lamp3^+^DCs in MM. Additional studies are necessary to assess these subsets in MM.

## 5 Role of DCs in MM-induced osteolytic bone disease

Most MM patients develop bone lesions due to excessive BM osteoclast (OC) formation, resulting from myeloma cell interactions with BM components (70, 71). This impacts OC and DC processes (72). Research shows that OC hyperactivity relates to DC dysfunction (73–76), stemming from their myeloid precursor relationship (77). Although some studies suggest OC precursors arise from monocyte/DC progenitors (MDPs), their exact nature is unclear (78). OC differentiation, or osteoclastogenesis, relies on macrophage colony-stimulating factor (M-CSF) and receptor activator of nuclear factor κB ligand (RANKL), activating T cells and DCs (45, 79). In MM, myeloma cells hinder normal osteoclastogenesis by recruiting, differentiating, and activating OC precursors in the BM (45, 73).

Additionally, interactions with immature DCs release cytokines like IL-6, increasing RANK-L and M-CSF production by stromal cells, osteoblasts, and myeloma cells (30, 45, 73, 75, 80). Elevated RANK-L in the BMME can convert DCs into OC-like cells via RANK-RANK-L interactions (76, 79). Supporting this, Tucci and colleagues confirmed that myeloma cells induce OC-like changes in moDCs (45). DCs also promote MM-induced osteolysis by overexpressing IL-17, produced by OCs and myeloma cells, as Th17 cells expand due to mature DCs (45, 81). This IL-17-rich environment enhances RANK-L activity, further stimulating osteoclastogenesis and osteolytic bone disease in MM and other cancers (81–84).

## 6 DC-based immunotherapies in MM

### 6.1 Current MM therapy

MM therapy advancements have significantly improved survival rates, with over 50% of patients surviving five years and 30% reaching ten years. Current standard treatment involves high-dose chemotherapy and hematopoietic stem-cell transplantation (HSCT). For newly diagnosed MM, a four-drug regimen including an anti-CD38 antibody, proteasome inhibitor, lenalidomide, and dexamethasone is widely used (85).

CAR T-cell therapy has revolutionized treatment for relapsed/refractory MM, mainly targeting BCMA (86, 87). Bispecific antibodies and immunocytokine therapies are emerging as promising options for relapsed cases. Bone-modifying agents like bisphosphonates and denosumab manage bone complications, while proteasome inhibitors and monoclonal antibodies remain essential (85).

For high-risk SMM, treatments such as daratumumab monotherapy or lenalidomide-based regimens delay disease progression (88, 89).

Many preclinical and clinical studies are investigating immunotherapeutic strategies, including DC-based vaccines, to enhance or restore the function of DCs in tumors (31, 32, 90).

### 6.2 DC-based vaccination

DC- based vaccines are promising in cancer immunotherapy. They activate tumor-specific T-cells to enhance cancer cell destruction (91). Their personalized nature allows customization based on individual tumor characteristics, offering a favorable safety profile with fewer immune-related adverse events than other immunotherapies (92). DC vaccines also induce long-lasting antitumor immunity, potentially reducing cancer recurrence and improving long-term outcomes. Additionally, they can convert “cold” tumors into “hot” ones, increasing T-cell infiltration and boosting the efficacy of subsequent treatments (91, 93, 94).

Despite these advantages, DC vaccines have shown limited clinical success due to challenges such as the immunosuppressive tumor microenvironment, manufacturing complexities, and suboptimal activation states of ex vivo-generated DCs (93). Most current vaccines rely on moDCs loaded with tumor-associated antigens (TAAs) or RNA (95). While moDCs are less efficient than natural DCs, they remain widely used due to their ease of production and scalability (96–98). Innovative strategies, such as loading DCs with a broad spectrum of antigens via tumor lysates or RNA electroporation, aim to enhance immune responses and minimize antigenic escape (99–102). However, as described above, mo-DCs derived from MM patients, exhibit a compromised functionality resembling tolerogenic DCs (64). This situation may hinder the efficacy of MM immunotherapy by fostering tolerance instead of immunity. To counteract this effect, strategies aimed at targeting tolerogenic DC-like dysfunction in MM—such as enhancing IL-12 production or inhibiting IL-10—could reflect tactics used against mregDCs in other cancers (103, 104).

Research is also exploring alternatives like DC-derived exosomes, which can carry cancer-specific antigens and stimulate robust immune responses while addressing some limitations of traditional DC vaccines (105, 106).

Despite the promise of DC-based vaccines, clinical results have varied, with only 5–15% of patients exhibiting significant immune responses and most experiencing relapses (94, 107). This limited efficacy stems from various immune escape and immunosuppressive mechanisms that foster therapy resistance.

### 6.3 Combinatory therapy

Consequently, considerable effort has been dedicated to transforming traditional DC vaccines into ‘next-generation’ versions that can counteract TME-related immunosuppression and bolster antitumor activity responses (108). Combination therapies have garnered significant interest in enhancing therapeutic effectiveness and tackling resistance by integrating DC vaccines with immune checkpoint inhibitors, CAR-T treatments, chemotherapy, and immunomodulatory agents (109, 110). Research indicates that modifying immune checkpoint molecules on DCs *in vitro* through siRNA, lentivirus, adenovirus, or CRISPR-Cas-9 boosts T-cell activation T cell activation (111). Similarly, Chu et al. demonstrated that combining DC vaccines with PD-L1 blockade effectively hinders tumor growth in MM models (112). Moreover, integrating DC vaccines with prior stem cell transplantation has led to an overall survival increase of nearly two years compared to patients who did not receive DC therapy (113, 114). Nonetheless, the optimal timing for DC immunotherapy continues to be debated, although evidence suggests that administering it following HSCT, when the disease burden is low, is preferable (115).

Efforts are ongoing to refine vaccine formulations, optimize antigen-loading techniques, and improve the *in vitro* generation of cDCs to mimic their natural counterparts better. These advancements aim to enhance the clinical effectiveness of DC-based vaccines and establish them as a cornerstone in cancer immunotherapy.

## 7 Discussion

This review explores DCs in MM, emphasizing their dual role in immune regulation and cancer progression. DCs, essential for antigen presentation and T-cell priming, are impaired in the MM tumor microenvironment (TME), contributing to immune tolerance and tumor growth. Understanding these dysfunctions and leveraging DCs as therapeutic targets remains a critical area of research.

DC dysfunction in MM involves reduced numbers, altered phenotypes, and impaired T-cell activation capacity. However, the molecular mechanisms driving these changes remain unclear, necessitating further investigation to identify therapeutic targets and restore DC function. Additionally, the heterogeneity of DC subsets—cDC1s, cDC2s, DC3s, pDCs, and moDCs—and their specific roles in MM progression require deeper exploration to develop targeted therapies.

The immunosuppressive nature of the MM TME poses significant challenges for DC-based therapies. Future strategies could include combination treatments targeting multiple immune pathways or enhancing DC-based vaccines’ efficacy through improved design and delivery methods. Personalized medicine approaches also promise to tailor DC vaccines to individual tumor profiles and immune statuses for better outcomes. This approach would require biomarker discovery and validation advancements to predict response to DC-based therapies and guide treatment decisions.

Another critical focus is long-term efficacy. Research should assess the durability of immune responses induced by DC therapies and explore maintenance strategies to sustain remission. Enhancing immunological memory against MM cells is key to improving survival rates.

Improving *ex vivo* DC manufacturing processes is vital for scaling up therapies. Alternatively, *in vivo*, approaches targeting DC activation directly in patients may overcome current limitations and improve treatment effectiveness.

In conclusion, while challenges remain, advancing our understanding of DC biology and MM pathogenesis could unlock the full potential of DC-based immunotherapies, offering hope for better outcomes in MM patients.
